# BTK‐Inhibition Enhances TLR‐7‐Mediated Interferon‐Alpha Production in pDCs by Blocking the Inhibitory BDCA‐2 Pathway

**DOI:** 10.1002/eji.202450985

**Published:** 2025-02-24

**Authors:** Laura Ceglarek, Ramona Gerhards, Vinicius Boldrini, Christian Wichmann, Anneli Peters, Edgar Meinl

**Affiliations:** ^1^ Institute of Clinical Neuroimmunology Biomedical Center and University Hospital Ludwig‐Maximilians‐Universität München Munich Germany; ^2^ Center for Human Immunology University of Zurich Zurich Switzerland; ^3^ Department of Transfusion Medicine Cell Therapeutics and Hemostaseology University Hospital, LMU Munich Munich Germany

**Keywords:** BTK‐i, TLR signaling, multiple sclerosis, pDCs, systemic lupus erythematosus

## Abstract

In pDCs, BTK‐inhibition (BTKi) blocks the IFN‐α production via TLR‐9, but not via TLR‐7. Upon TLR‐7 stimulation, BTKi enhances the production of IFN‐α by blocking the inhibitory BDCA‐2 pathway. This might explain partially the failure of BTKi in SLE and is of interest for BTKi trials in multiple sclerosis.

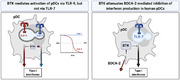

**Abbreviations**
BTK: Bruton's tyrosine kinase
BTKi: Bruton's tyrosine kinase inhibitors
IFN: interferon
MS: multiple sclerosis
pDCs: plasmacytoid dendritic cells
SLE: systemic lupus erythematosus
TLR: Toll‐like receptor

## 1 Notes and Insights

Bruton's tyrosine kinase (BTK) is an integral component of the B cell receptor (BCR)‐signalosome and participates in Fc‐ and Toll‐like receptor (TLR) signaling [1]. While a role for BTK is well‐established in the activation of immune cells, BTK's function in inhibitory pathways is unclear. BTK inhibitors (BTKi) are explored in different autoimmune diseases with disparate results. In systemic lupus erythematosus (SLE), BTKi unexpectedly failed [2], while BTKi is promising in multiple sclerosis (MS) [3]. These differing results may in part be due to different immunological mechanisms underlying pathogenesis related to distinct roles of type 1 interferons. While IFN‐β is an established therapy in relapsing MS and the interferon‐alpha/beta receptor (IFNAR) signaling on myeloid cells limits autoimmunity in the CNS [4], IFN‐α drives pathogenesis in SLE. After activation via TLR‐7 and TLR‐9, pDCs become major producers of IFN‐α and thus contribute to autoimmune inflammation [5].

**FIGURE 1:**
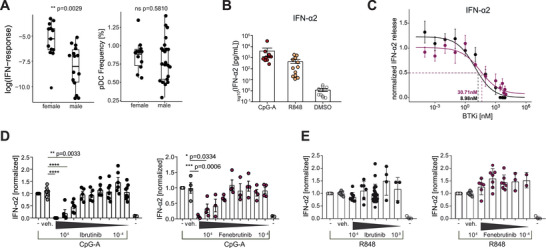
BTKi reduces TLR‐9‐, but not TLR‐7 mediated activation of human pDCs. (A) IFN‐responsiveness of enriched pDCs and pDC frequencies based on the sex (male *n* = 22; female *n* = 13). The interferon response index was determined as described in the supplement and tested using a two‐tailed *t*‐test. (B) Purified pDCs (*n* = 10) were stimulated with TLR agonists and the release of IFN‐α2 was determined. (C) Purified pDCs (*n* = 12) were treated with Ibrutinib (black) or Fenebrutinib (purple) for 1 h prior to stimulation with CpG‐A (2.5 µg/mL). IC_50_ values were determined by logistic regression using a four‐parameter log‐logistic model. (D, E) pDCs (*n* = 17) were treated with the BTKi for 1 h prior to stimulation with (D) CpG‐A (2.5 µg/mL) and (E) R848 (10 µg/mL). Data represent the mean ± SEM of independent experiments with each donor. For (B, C, E) data were analyzed by one‐way ANOVA with Tukey's post hoc tests.

In this study, we analyzed the effect of BTKi (Fenebrutinib and Ibrutinib) on the IFN‐α production of pDCs via TLR‐7 or TLR‐9, and on the inhibitory BDCA‐2 pathway. Binding and subsequent internalization of BDCA‐2, a C‐type lectin exclusively expressed on human pDCs, inhibits interferon production [6]; however, the exact composition of the signalosome mediating this cascade is not fully elucidated. We observed that pDCs of females produced more IFN‐α upon TLR‐7‐ligation (Figure 1A). This might arise from the effects of testosterone or incomplete inactivation of the X‐linked TLR‐7 gene [7]. Activation of pDCs via TLR‐9 (using CpG) and TLR‐7 (using R848) induced the release of IFN‐α2 (Figure 1B; Figure S1), reflecting the overall IFN‐α production (Figure 1C; Figure S1A–C and references therein). BTKi reduced the release of IFN‐α2 after stimulation via TLR‐9, but not via TLR‐7 (Figure 1C–E). Which kinase can substitute for BTK in the TLR7 pathway in pDCs remains to be investigated. In contrast to the observed effects on pDCs, BTKi blocked the activation of B cells by both TLR‐7 and TLR‐9 (Figure S2A–D), consistent with previous reports [8]. Furthermore, BTKi abrogated TLR‐driven plasmablast differentiation but did not affect differentiated plasmablasts (Figure S2E,F).

**FIGURE 2:**
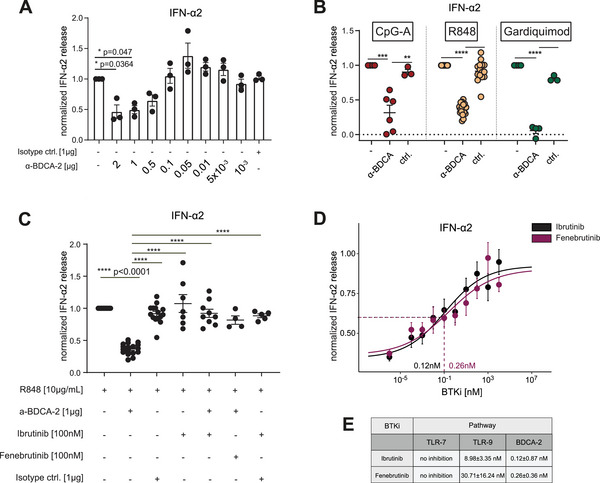
BTK‐i abrogates the IFN‐attenuating effect of BDCA‐2 in pDCs. (A) pDCs (*n* = 3) were stimulated with R848 and an agonistic mAb to BDCA‐2 or isotype control Ab and the secreted IFN‐α2 was determined. (B) pDCs (*n* = 17) were stimulated with the indicated TLR ligands in the presence of no Ab (‐), the mAb to BDCA‐2 (10 µg/mL), or a control mAb (10 µg/mL). (C) pDCs were stimulated with R848. An anti‐BDCA‐2 mAb, a control mAb, Ibrutinib (100 nM), and Fenebrutinib (100 nM) were added as indicated. (D) pDCs (*n* = 13) were treated with Ibrutinib (black) or Fenebrutinib (purple) for 1 h prior to stimulation with R848 (10 µg/mL) and the addition of anti‐BDCA‐2 mAb (10 µg/mL). Supernatants were collected after 15 h and analyzed for IFN‐α2. (E) IC_50_ values for BTKi inhibiting TLR‐7, TLR‐9, and BDCA‐2 mediated activation. IC_50_ were determined by 4‐parameter‐logistic modeling.

Next, we analyzed whether BTKi affects the BDCA‐2 pathway. Engagement of BDCA‐2 reduced IFN‐α production by pDCs after stimulation with different TLR ligands (Figure 2A,B), extending previous observations [6]. Since the induction of IFN‐α by R848 was not blocked by BTKi (Figure 1E), we used this system to analyze the role of BTKi in the BDCA‐2 pathway. BTKi abrogated the BDCA‐2‐mediated inhibition of IFN‐α production (Figure 2C,D). Intriguingly, the blocking of BDCA‐2 signaling was more sensitive to BTKi than the corresponding inhibition of activation via TLR‐9 in pDCs (Figure 2E).

BDCA‐2 engagement has been reported to induce the formation of a signaling complex resembling the BCR signalosome and it was proposed that BTK may participate in BDCA‐2 signaling [9], although experimental evidence was lacking. Our experiments established that BTKi blocks the inhibition of IFN‐α via BDCA‐2. We compared the BTKi Ibrutinib and Fenebrutinib throughout. While Fenebrutinib is specific for BTK, Ibrutinib also targets other TEC family kinases. Thus, our experiments indicate that BTK is the only TEC family kinase required for BDCA‐2 signaling. Further direct mechanistic evidence of BTK phosphorylation upon BDCA‐2 engagement and downstream signaling, for example, via IRF‐7 should be elaborated.

Our finding that BTKi block the BDCA‐2 pathway thereby enhancing IFN‐α production might contribute to the explanation of why BTKi failed unexpectedly in SLE [2]. First, IFN‐α is commonly regarded to drive SLE, and in line with this, females produce more IFN‐α in response to TLR‐7 engagement (Figure 1 and [7]) and develop SLE far more frequently than men. Second, stimulating BDCA by agonistic antibodies is a promising therapy in SLE [10]. In mouse models of SLE, BTKi was successful, but as BDCA‐2 is exclusively found in humans, the enhancement of IFN‐α production by blocking the BDCA‐2 pathway with BTKi could not be mirrored in those models.

Collectively, our findings contribute to further understanding the failure of BTKi in SLE and might be of relevance for ongoing clinical trials in MS.

## Conflicts of Interest

The authors declare no conflicts of interest.

### Peer Review

The peer review history for this article is available at https://publons.com/publon/10.1002/eji.202450985.

## Supporting information



Supporting Information

## Data Availability

Detailed protocols and computational analysis pipelines of this study as well as data are available from the corresponding author upon request.
