# Supplementary material for: BTK‐Inhibition Enhances TLR‐7‐Mediated Interferon‐Alpha Production in pDCs by Blocking the Inhibitory BDCA‐2 Pathway
Source: Eur J Immunol. 2025 Feb 24;55(2):e202450985. doi: 10.1002/eji.202450985 (PMC11848701; doi:10.1002/eji.202450985)
Supplement: Supplementary file 1 — Supporting Information [file EJI-55-e202450985-s001.pdf]

Supporting information for

"BTK-inhibition enhances TLR-7 mediated interferon-alpha production in pDCs by blocking the inhibitory BDCA-2 pathway"

Laura Ceglarek, Ramona Gerhards, Vinicius Boldrini, Christian Wichmann, Anneli Peters and Edgar Meinl

Materials and Methods

Cell isolation and culture

Human PBMCs were isolated from leukoreduction system chamber (LRS) buffy coats provided by the Transfusion Medicine Department of the LMU Clinic Munich (Germany) and sourced from the ATMZH platelet donation bank as previously described [1]. Blood donors were healthy men and women aged between 24 and 65 years (mean age 45 years, male:female (m:f) ratio 32:16) who tested negative for HIV, hepatitis B virus, and hepatitis C virus. Peripheral blood mononuclear cells (PBMCs) were obtained by Pancoll density gradient centrifugation (PanBiotech, Aidenbach, Germany). pDCs were isolated by using the human Plasmacytoid Dendritic Cell Isolation Kit II (Miltenyi Biotec, Bergisch Gladbach, Germany) and the EasySep™ Human Plasmacytoid DC Enrichment Kit (STEMCELL Technologies, Vancouver, Canada) according to the manufacturers' instructions. pDC purity routinely exceeded 85% as determined by BDCA-2 expression. Similarly, human B cells were enriched by immunomagnetic negative selection (EasySep™ Human B Cell Enrichment Kit, STEMCELL Technologies). B cell purity was determined by CD19 expression and routinely exceeded 97%. Enriched human pDCs and B cells were cultured in RPMI 1640 medium supplemented with 10% fetal bovine serum (FBS), 2 mM L-Glutamine, 1 mM Sodium pyruvate, 100 U/mL penicillin, 100 µg/mL streptomycin and 1% non-essential amino acids.

Oligodeoxynucleotides and in vitro stimulation reagents

The following unmethylated CpG oligodeoxynucleotides of types A, B and C were all purchased from InvivoGen (San Diego, CA) (lower case letters indicate phosphorothioate linkages and capital letters indicate phosphodiester linkages at the 3' end of the corresponding base): ODN2216 (CpG-A), 5'-ggGGGACGA:TCGTCgggggg-3'; ODN2006 (CpG-B) 5'-tcgtcgttttgtcgttttgtcgtt-3'; ODN2395 (CpG-C), 5'-tcgtcgttttcggcgc:gcgccg-3'. CpG-A, CpG-B and CpG-C stimulate TLR-9. R848 stimulates both TLR-7 and TLR-8; since B cells and plasma cells do not express TLR-8 [2], R848 is considered to activate TLR-7 in pDCs and B cells. Ibrutinib (PCI-32765, free base) was purchased from LC Laboratories, Woburn, MA, USA and Fenebrutinib (GDC-0853) was purchased from MedChemExpress, South Brunswick, NJ, USA.

Overview of the TLR ligands used in this study

TLR-ligand	concentration	assay	Manufacturer
Resiquimod (R848)	10 µg/mL	pDC stimulation	InvivoGen, San Diego, CA, USA
Gardiquimod	0.175 µg/mL	pDC stimulation	Sigma-Aldrich, St. Louis, MO, USA
Cdp14b	2.5 µg/mL	pDC stimulation	Sigma-Aldrich, St. Louis, MO, USA
rhIL-2	1,000 IU/mL	B-cell stimulation	R&D Systems, Minneapolis, MN, USA

Overview of the antibodies used in this study

antigen	fluorophore	clone	Manufacturer (Cat.No)
BDCA-2 (CD303)	FITC	AC144	Miltenyi Biotec, 130-113-754
CD19	FITC	HIB19	Biolegend, 302256
CD19	PE-Cyanine7	HIB19	Biolegend, 302216
CD38	PE	HIT2	Biolegend, 303505
CD27	PE-Cy7	O323	eBioscience, 25-0279-42

Human pDC stimulation assays

Preparation of PBMC and purification of B cells and pDCs is described above. Isolated pDCs were seeded into 96-well culture plates (0.2×10^5 cells/mL). Cells were pre-treated with vehicle (0.2% dimethyl sulfoxide; DMSO) or the corresponding inhibitors for 60 min before stimulation. pDCs were stimulated with CpG-A (ODN2216, 2.5 µg/mL), CpG-B (ODN2006, 2.5 µg/mL), Resiquimod (R848, 10 µg/mL), Gardiquimod (0.175 µg/mL) or Cdp14b (2.5 µg/mL) for 15–72 h. Anti-BDCA-2 mAb (CD303 mAb, 10 µg/mL, clone AC144, Miltenyi Biotec) or IgG1κ mAb (purified NA/LE Mouse IgG1 κ isotype control, 10 µg/mL, clone 107.3RUO, BD Pharmingen, San Diego, CA, USA) were added shortly before stimulation. IFN-α (pbl Assay Science, Piscataway, NJ, USA), IFN-α2 (IFNA2 DuoSet ELISA, R&D Systems, Minneapolis, MN, USA) and sBCMA (BCMA/TNFRSF17, DuoSet ELISA, R&D Systems) were detected utilizing the respective ELISA kits according to the manufacturer's instructions. Interferon response index was calculated based on the IFN-α2 released measured in the cell culture supernatant corrected for the absolute amount of pDCs seeded. The absolute amount of pDCs was determined by counting total seeded cells using an automated cell counter and correcting that cellcount with the percentage of the BDCA-2$^+$ cells as determined by flow cytometry.

Human B cell stimulation assays

Enriched B cells were seeded into 24-well culture plates (3×10^5 cells/mL). Cells were pre-treated with vehicle (0.2% DMSO) or the corresponding inhibitors for 60 min before stimulation. B cells were stimulated with CpG-A (ODN2216, 2.5 µg/mL), CpG-B (ODN2006, 2.5 µg/mL), CpG-C (ODN2395,

2.5 µg/mL), Resiquimod (R848, 1 µg/mL) and recombinant IL-2 (1,000 IU/mL) or Gardiquimod (0.175 µg/mL) for 4 days as previously described [3]. IgG, IgA and IgM (Mabtech, Nacka Strand, Sweden) and sTACI (TACI/TNFRSF13B DuoSet ELISA, R&D Systems) were detected in the cell culture supernatants using the appropriate ELISA kits according to the manufacturer's instructions.

Plasmablast differentiation of human B cells

To induce differentiation into plasmablasts, human PBMCs were seeded on 24-well plates (1×10^6 cells/mL). Cells were pre-treated with BTK-i or vehicle and stimulated with R848 (1 µg/mL) and recombinant IL-2 (1,000 IU/mL) for 5 days and 7 days, respectively as previously described [3]. Plasmablast differentiation was determined by surface expression of CD38 and CD27.

Statistical analysis

Flow Cytometry data was analyzed using FlowJo (10.7.1, BD, Franklin Lakes, NJ, USA). Statistical analyses were performed using GraphPad Prism 7 and 9.0.2 (GraphPad Software Inc., La Jolla, CA, USA). Dose-response modelling was carried out in R (version 4.0.4) using the drc package [4] utilizing a four-parameter log-logistic model. Tukey's multiple comparisons test was used for comparisons of two independent groups. Significance was assessed by non-parametric methods unless specified otherwise. All tests were two-sided. *P*-values of differential expression within the scRNA-seq dataset were adjusted for multiple comparison using the Benjamini-Hochberg method. Spearman's rank correlation was used to assess associations of numeric variables. Data were visualized with *ggplot2* (3.3.5), *corrplot* (0.90), and *ggpubr* (0.4.0). $P < 0.05$ was considered to indicate statistical significance.

Supplementary Figures

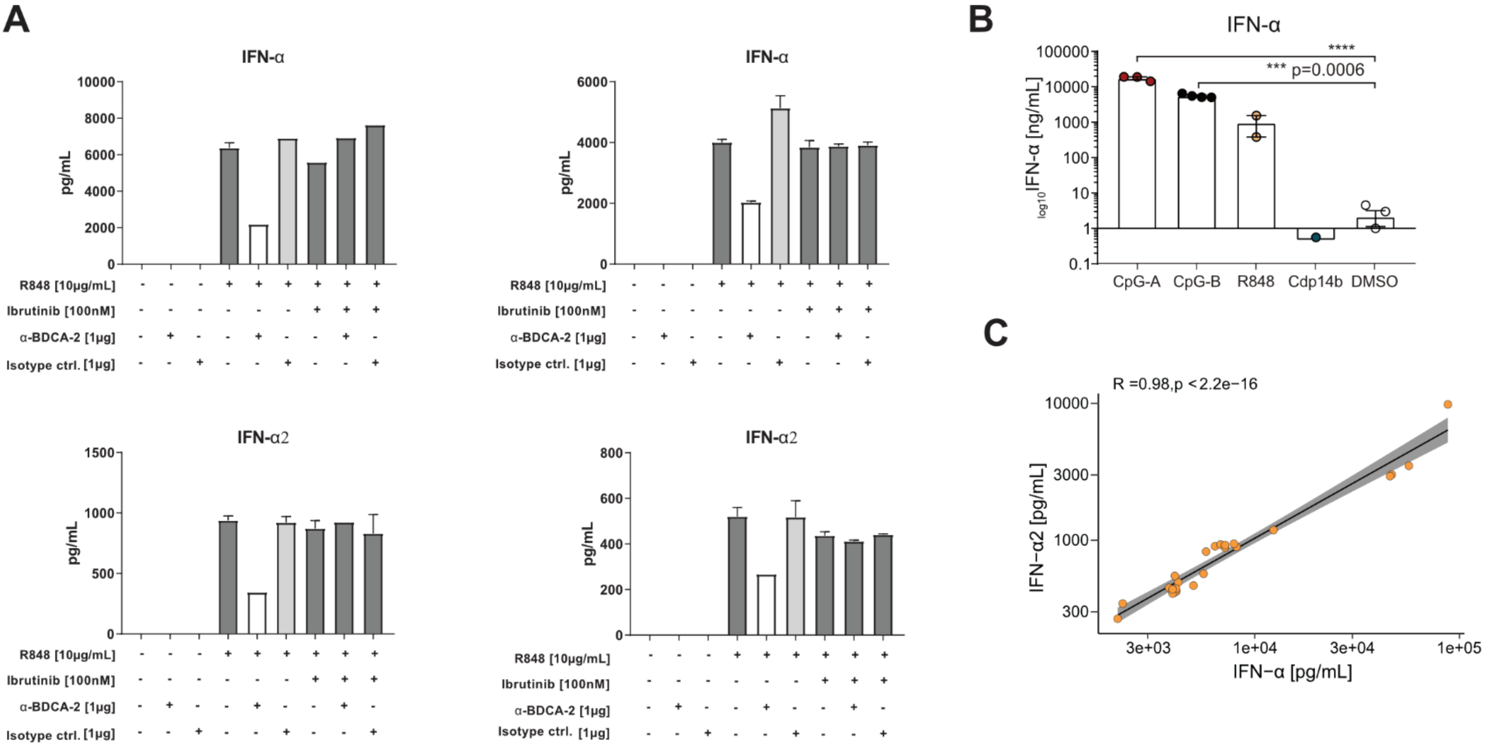

Supplementary Figure 1: Activation of pDCs via TLR-ligands. Secretion of IFN-α2 is representative of the whole IFN-α level produced by human pDCs.

A) pDC were stimulated as indicated and the amount of released total IFN-a and of IFN-a2 was determined after 24 hours. Data from two representative experiments showing that IFN-α2 (bottom) is representative of the whole IFN-α secretion (top) of pDCs after TLR-ligation. **B**) Purified pDCs (n=4) were stimulated with TLR agonists and the release of IFN-α was determined. TLR ligands induced differential IFN- α production as previously reported [5]. The TLR8 agonist Cdp14b did not induce any response in pDC, which is in line with the lack of TLR8 expression in pDC [2]. Therefore, we conclude that the effects of R848, which activates both TLR7 and TLR8 are based on TLR7 activation. **C**) Correlation of IFN-α2 and IFN-α detected in the cell culture supernatant as previously reported [6]. Spearman correlation coefficient is shown and determined as described in the methods section of values from A). Unless otherwise specified, supernatants were collected after 24 h of *in vitro* culture. Not all conditions were analysed for each donor. Data represent the mean ± SEM of independent experiments with each donor

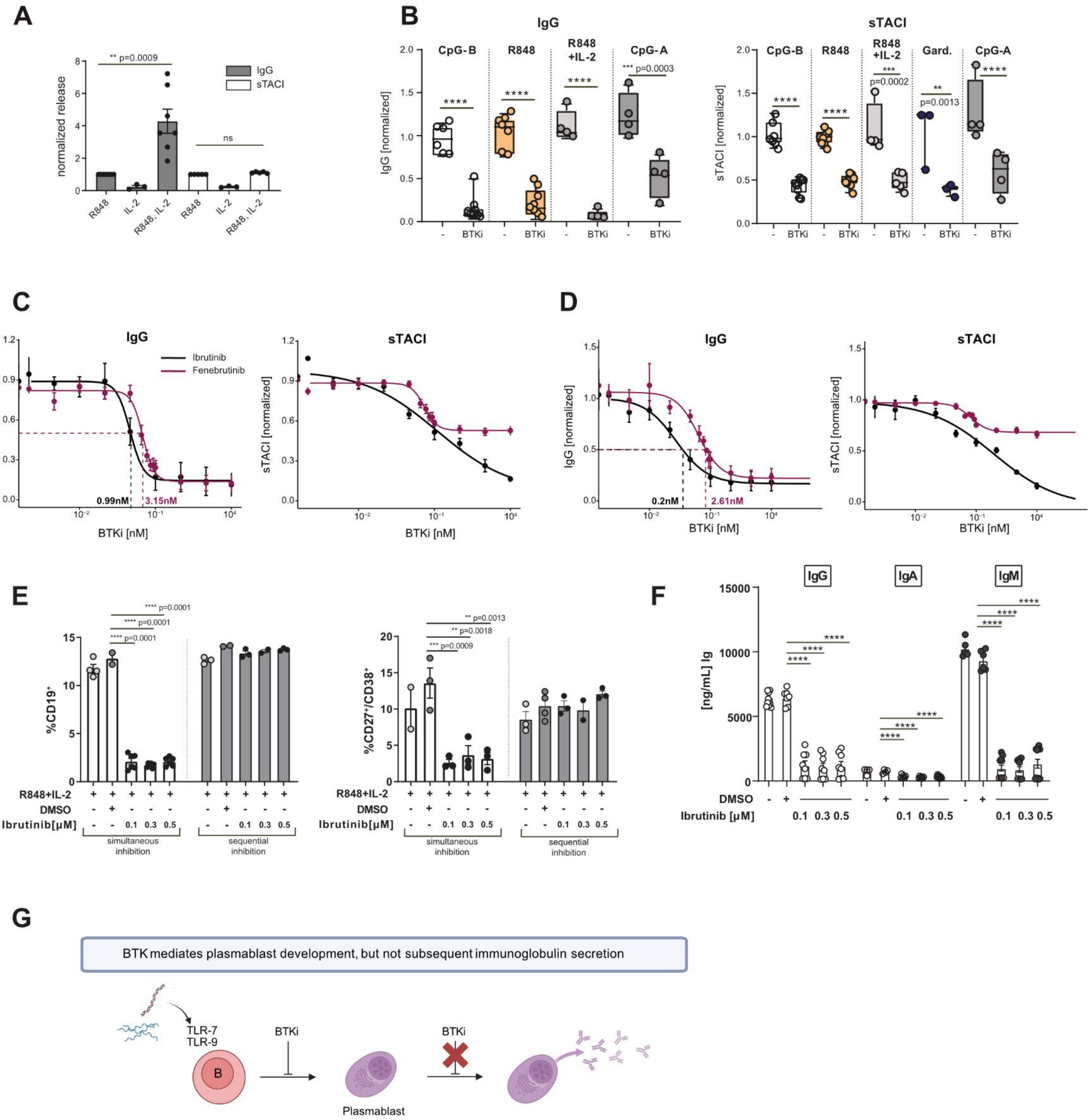

A
normalized release
** p=0.0009
IgG
sTACI
ns
R848
IL-2
R848, IL-2
B
IgG
CpG-B
R848
R848 +IL-2
CpG-A
*** p=0.0003
IgG [normalized]
sTACI
Gard.
*** p=0.0002
** p=0.0013
sTACI [normalized]
BTKi
C
IgG
Ibrutinib
Fenebrutinib
IgG [normalized]
0.99nM
3.15nM
BTKi [nM]
sTACI
sTACI [normalized]
D
0.2nM
2.61nM
E
**** p=0.0001
%CD19⁺
R848+IL-2
DMSO
Ibrutinib[µM]
simultaneous inhibition
sequential inhibition
** p=0.0013
** p=0.0018
*** p=0.0009
%CD27⁺/CD38⁺
F
IgG
IgA
IgM
[ng/mL] Ig
DMSO
Ibrutinib [µM]
G
BTK mediates plasmablast development, but not subsequent immunoglobulin secretion
TLR-7
TLR-9
B
BTKi
Plasmablast

Supplementary Figure 2: BTK-i reduces TLR-7- and TLR-9-mediated activation of B cells. B cells from healthy donors were purified and cultured with the indicated stimuli and BTK-i. **A**) IgG and sTACI release from purified B cells (n=11) were determined after 4 days of stimulation. IL-2 boosts IgG (grey) production by enriched human B cells after TLR-7 engagement by R848 but not sTACI (white) secretion. The data were analyzed by 1-way ANOVA with Tukey's post-hoc tests. **B**) Ibrutinib (300 nM) was added to enriched B-cells 1 h prior to stimulation with CpG-B (2.5 µg/mL), CpG-A (2.5 µg/mL), R848 (1 µg/mL), R848 (1 µg/mL) + IL-2 (25 ng/mL), or Gardiquimod (1.75 µg/mL). Released amounts of IgG and sTACI were determined. Data represent the mean ± SEM (n = 8 independent experiments with 8 different donors). Secreted proteins were normalized for each individual donor to the baseline level of protein secretion upon TLR-stimulation without any inhibitor present. The data were analyzed by 1-way ANOVA with Tukey's post-hoc tests (**** denotes a significance level of $P \leq 0.0001$) **C, D)** Ibrutinib (black) and Fenebrutinib (purple) were added in increasing doses to B cells stimulated with CpG-B (**C**) or R848 (**D**). Data represent the mean ± SEM (n = 5 independent experiments with 5 different donors). Not all conditions were analyzed for each individual donor. Dose-response and IC_{50} values were determined by 4-parameter-log-logistic modelling of the normalized dose response data. **E**) PBMCs were stimulated for 7 d with R848 (2.5 µg/mL) and IL-2 (25 ng/mL). The BTK inhibitor Ibrutinib was added simultaneously to TLR-stimulation (white) or sequentially added after 7d (grey). The fraction of $CD19^+$ B cells (left) and $CD19^+CD27^+CD38^+$ plasmablasts (right) was determined by flow cytometry. Data represent the mean ± SEM (n = 2 independent experiments with 2 different donors; *P*-values determined by Tukey's multiple comparison test). **F**) PBMC were stimulated with R848+IL-2 for seven days as in E). Secretion of IgG, IgA, and IgM was determined by ELISA in the. Data represent the mean ± SEM (n = 2 independent experiments with 5 different donors; *P*-values determined by Tukey's multiple comparison test). **G)** Cartoon illustrating the data shown in A-F.

References

1. **Hoffmann FS, Kuhn P-H, Laurent SA, Hauck SM, Berer K, Wendlinger SA, Krumbholz M, *et al.*** The Immunoregulator Soluble TACI Is Released by ADAM10 and Reflects B Cell Activation in Autoimmunity. *J. Immunol.* 2015; **194**:542–552.DOI: 10.4049/jimmunol.1402070.

2. **Hornung V, Rothenfusser S, Britsch S, Krug A, Jahrsdörfer B, Giese T, Endres S, *et al.*** Quantitative Expression of Toll-Like Receptor 1–10 mRNA in Cellular Subsets of Human Peripheral Blood Mononuclear Cells and Sensitivity to CpG Oligodeoxynucleotides. *J. Immunol.* 2002; **168**:4531–4537.DOI: 10.4049/jimmunol.168.9.4531.

3. **Pinna D**, **Corti D**, **Jarrossay D**, **Sallusto F**, **Lanzavecchia A**. Clonal dissection of the human memory B cell repertoire following infection and vaccination. *Eur. J. Immunol.* 2009; **39**:10.1002/eji.200839129.DOI: 10.1002/eji.200839129.

4. **Ritz C**, **Baty F**, **Streibig JC**, **Gerhard D**. Dose-Response Analysis Using R. *PLOS ONE*. 2015; **10**:e0146021.DOI: 10.1371/journal.pone.0146021.

5. **Kerkmann M, Rothenfusser S, Hornung V, Towarowski A, Wagner M, Sarris A, Giese T, *et al.*** Activation with CpG-A and CpG-B Oligonucleotides Reveals Two Distinct Regulatory Pathways of Type I IFN Synthesis in Human Plasmacytoid Dendritic Cells. *J. Immunol.* 2003; **170**:4465–4474.DOI: 10.4049/jimmunol.170.9.4465.

6. **Szubin R**, **Chang WLW**, **Greasby T**, **Beckett L**, **Baumgarth N**. Rigid Interferon-α Subtype Responses of Human Plasmacytoid Dendritic Cells. *J. Interferon Cytokine Res.* 2008; **28**:749–763.DOI: 10.1089/jir.2008.0037.
